# A meta-analysis on genetic variability of RT/HBsAg overlapping region of hepatitis B virus (HBV) isolates of Bangladesh

**DOI:** 10.1186/s13027-019-0253-6

**Published:** 2019-11-07

**Authors:** Md. Golzar Hossain, Keiji Ueda

**Affiliations:** 1 Division of Virology, Department of Microbiology and Immunology, Graduate School of Medicine, Osaka University, Osaka, Japan; 2 Department of Microbiology and Hygiene, Bangladesh Agricultural University, Mymensingh, Bangladesh

**Keywords:** Hepatitis B virus (HBV), Serotype, Genotype, HBsAg escape mutations, Drug-resistant mutations, Bangladesh

## Abstract

**Background and aim:**

Hepatitis B caused by HBV is a serious public health hazard prevalent worldwide including Bangladesh. Few scattered molecular studies of HBV have been reported in Bangladesh. This study aimed to analyze the genetic variability of RT/HBsAg overlapping region of HBV isolates of Bangladesh and determination of correlation among the genotype/serotype and HBsAg escape and/or drug-resistant mutations.

**Methods:**

A total of 97 complete HBsAg sequences of Bangladeshi HBV isolates from 2005 to 2017 from NCBI GenBank were extracted and analyzed using several HBV bioinformatics tools such as Geno2pheno-HBV, HBV Serotyper, HIV-Grade:HBV-Tool, and CLC sequence viewer.

**Results:**

The prevalence of genotypes A, C, and D are 18, 46 and 35% which correspond to serotype *adw*, *adr*, and *ayw,* respectively. The prevalence of HBsAg escape mutations is 51% and most of which (62%) are found in the genotype D followed by 32% in genotype C and 6% in genotype A. Interestingly most (24/36) of the sequences of HBsAg escape mutations contained 128 V mutant which all belongs to only serotype *ayw3* (Genotype D). Prevalence of drug-resistant mutations is ~ 11%, most of which are from genotype C (63.64%) and D (36.36%). Lamivudine resistant mutations were found in ~ 11% of sequences followed by Telbivudine 10% and Adefovir 3% where Tenofovir showed susceptibility to all 97 sequences. Moreover, 7 among of 97 sequences showed both HBsAg and drugs resistant mutations and none of them are found due to the same nucleotide substitutions.

**Conclusion:**

There is a strong correlation among the genotype/serotype and HBsAg escape and/or drug-resistant mutations. This meta-analytical review will be helpful for genotype-serotype prediction by PCR-based diagnosis and development of vaccine and/or diagnostic kits, and the treatment against HBV infection in the future.

## Introduction

Hepatitis B is caused by one of the smallest enveloped DNA viruses known as HBV under the family of hepadnaviridae. HBV can transmit vertically (mother to baby) and by horizontally such as sexual contact, sharing needles, syringes, razors, and blood transfusion, etc., [1, 2]. HBV causes both acute and chronic infections. Liver failure may occur in some cases of acute HBV infection which leads to sudden death. About 15–25% people of the chronically HBV infected patients may develop liver cirrhosis followed by hepatocellular carcinoma (HCC). However, liver cirrhosis and HCC may become more worsen due to coinfection with the hepatitis delta virus (HDV) and HBV because of using HBV envelope proteins by HDV for replication [3].

HBV genome is a partially double-stranded circular DNA of 3.2 kb in size. Four open reading frames (ORFs): P, C, S, and X are encoded by the viral genome. Small HBsAg envelope protein produced by the common C-terminal domain of ORF S, composed of 226 amino acids entirely overlapped with reverse transcriptase (RT) domain of viral polymerase (Pol) protein produced by ORF P [4]. There are four major subtypes (Serotypes) of HBsAg; *adw*, *ayw*, *adr*, and ayr defined by a common “a” determinant and two mutually exclusive determinants pairs, d/y and w/r at the positions 122 and 160, respectively [5, 6]. Clinical diagnosis of HBV infection is primarily based on the detection of HBsAg whereas recombinant HBsAg is used as a vaccine [7, 8]. Because of lack of Pol proofreading activity, mutation frequently occurs of HBV genome including the ORF for HBsAg protein that leads to HBsAg detection failure (diagnostic escape) by conventional routine diagnostic HBsAg ELISA and vaccination failure (Vaccine escape) [9–14]. On the other hand, many drug resistant mutations in the RT domain of Pol has been identified. The HBsAg coding sequence completely overlapped on the RT domain of HBV polymerase laying from amino acid position rt10 to rt234 [9, 11, 15, 16]. The RT/HBsAg overlapping region contained majority of the nucleotide conservation and the YMDD (amino acids rt203 to rt206) motif on the RT domain is very important for its function also laid within HBsAg coding region (*HBsAg* nt582 to nt593; Accession No.: JQ514509.1) [15, 17]. Moreover, most of the mutants resistant to anti-HBV drugs are within this RT/HBsAg overlapped sequence [18]. Higher rate of mutations in the RT/HBsAg overlapping region correlates with the lower level of serum HBD DNA and HBsAg which attract the researchers to focus on this topic [19].

Hepatitis B is prevalent worldwide including the WHO South-Asian region with a tentatively 2% of infection among the general people. It is also prevalent in Bangladesh ranging from 0.8 to 6.2% [20–22]. Few molecular analysis of HBV genetic analyses in Bangladesh have been performed with a narrow time period with limited regions [22–24]. These studies were limited to a specific region and or institute and some of them has no drug-resistant analysis or serotype (subtype) determination or HBsAg escape mutations analysis. Moreover, there is no report so far showing the relationship among genotype, serotype (subtype), HBsAg escape, and/or drug-resistant mutations. Therefore, the aim of the meta-analytical review is to determine the genetic variability of RT/HBsAg overlapping region of HBV isolates of Bangladesh and assessment of correlation among the genotype/serotype and HBsAg escape and/or drug-resistant mutations along with their prevalence determination.

## Methods

### Collection and processing of HBV sequences from NCBI database

A total of 97 complete coding sequences of HBsAg were collected from the NCBI GenBank by searching as HBV, Bangladesh under the nucleotide database (Additional file 1). The search results were as “Hepatitis B virus” [Organism] OR HBV [All Fields] AND Bangladesh [All Fields]. However, we also searched with other keywords but no additional sequences were found. We have taken only the complete HBsAg cds excluding the partial cds. Partial cds would not explain the exact number of mutations in the HBsAg region overlapped with RT. These HBsAg sequences were from 2005 to 2017 as dated either by sample collection or submission to the GenBank. The nucleotide sequences copied from NCBI were processed in the CLC Sequence viewer (http://www.clcbio.com) for further use.

### Determination of serotype (subtype) and genotype

The genotypes of all these HBsAg sequences were re-determined using the online bioinformatics tool Geno2pheno-HBV (https://hbv.geno2pheno.org). Serotype of some HBsAg sequences submitted from Bangladesh into NCBI GenBank was not mentioned previously and we determined the serotype of those sequences by online HBV Serotyper (http://hvdr.bioinf.wits.ac.za/serotyper/) [25].

### Analysis of HBsAg escape and drug-resistant mutation

The HBsAg escape and drug-resistant mutational analyses were performed using Geno2pheno-HBV (https://hbv.geno2pheno.org) and HIV-Grade:HBV-Tool (https://www.hiv-grade.de/cms/grade/explanations/hbv-tool/) [26, 27]. The Geno2pheno-HBV provides the HBsAg escape mutation with the mode of action such as vaccine, detection, and therapy escape whereas HIV-Grade:HBV-Tool concludes only as typical mutations for HBsAg escape detected.

### Statistical analysis

Statistical analysis was performed using JavaScript-STAR version BETA-E. To determine the *p*-value among different parameters, Chi-square test was performed and the significance level was considered as 0.05.

## Results

### Prevalence of genotypes and serotypes (subtypes)

**Fig. 1:**
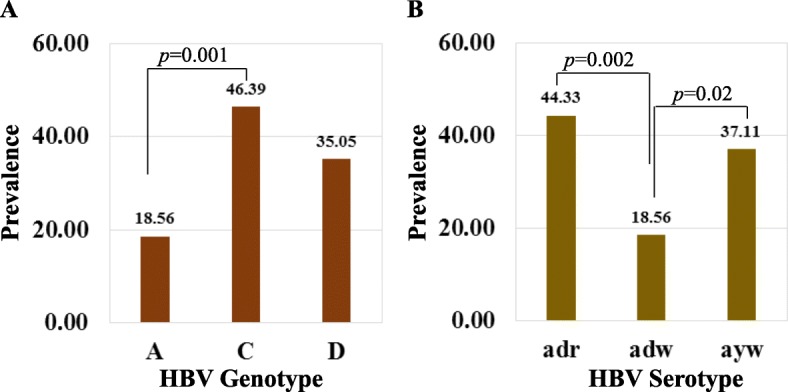
Prevalence of HBV genotype and serotype**.** Ninety-seven complete small HBsAg coding sequences of Bangladeshi HBV isolates of year, 2005–2017 were collected from NCBI GenBank. The HBV genotype and serotype were re-determined by using the bioinformatics tool followed by an analysis of their overall prevalence of genotype (**a**) and serotype (**b**) circulating in Bangladesh. Chi-square test was performed to determine the statistical significance level and only the *p-*value greater than 0.05 was mentioned

The HBV genotype and serotype prevalence among the 97 complete HBsAg coding sequences from the NCBI GenBank during 2005–2017 deposited as Bangladeshi HBV isolates were determined. Results demonstrated that only three genotypes, A, C, and D are circulating in Bangladesh with 7 subgenotypes A1, A2, C1, C2, D1, D2, and D3 (Fig. 1a). The predominant genotype is C (46.39%) followed by D (35.05%) and A (18.56%). Serotyping analysis showed that the most common subtype is *adr* (44.33%). The prevalence of serotype *ayw* (*ayw1*, *ayw2*, and *ayw3*,) and *adw* (*adw2*) is 37.11 and 18.56%, respectively. However, within these 97 sequences, there is no report of serotype *ayr* (Fig. 1b).

### Relationship among the genotypes and subtypes

**Fig. 2:**
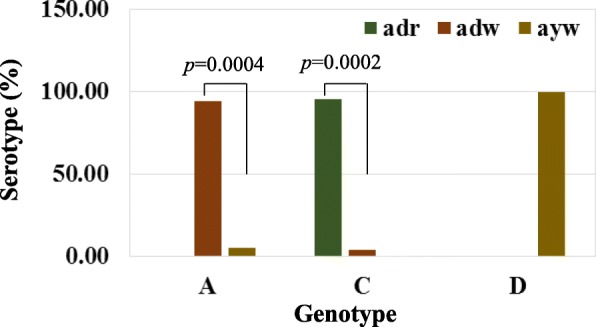
Distribution of HBV genotype among the serotype. Relationship of HBV genotype and serotype of Bangladeshi HBV isolates from 2005 to 2017 was determined. Chi-square test was performed to determine the statistical significance level and only the *p-*value greater than 0.05 was mentioned

We wanted to know whether there is any relationship among the HBV genotypes and serotypes (subtypes) of HBV isolates of Bangladesh during the long times courses from 2005 to 2017. Among the 45 of genotype C sequences, 43 (95.56%) are under subtype *adr* and only 2 (4.44%) are of *adw*. The 17 (94.44%) out of total 18 genotype A is under the serotype *adw* and only 1 (5.56%) is under *ayw*. In case of genotype D, all 34 (100%) are under the serotype *ayw* (Fig. 2). These results strongly suggested that there is a significant relationship between HBV genotypes and serotypes (subtypes) of Bangladeshi isolates such as serotypes *adr*, *adw*, and *ayw* belongs to genotypes C, A, and D, respectively.

### Analysis and prevalence of HBsAg escape mutations

**Fig. 3:**
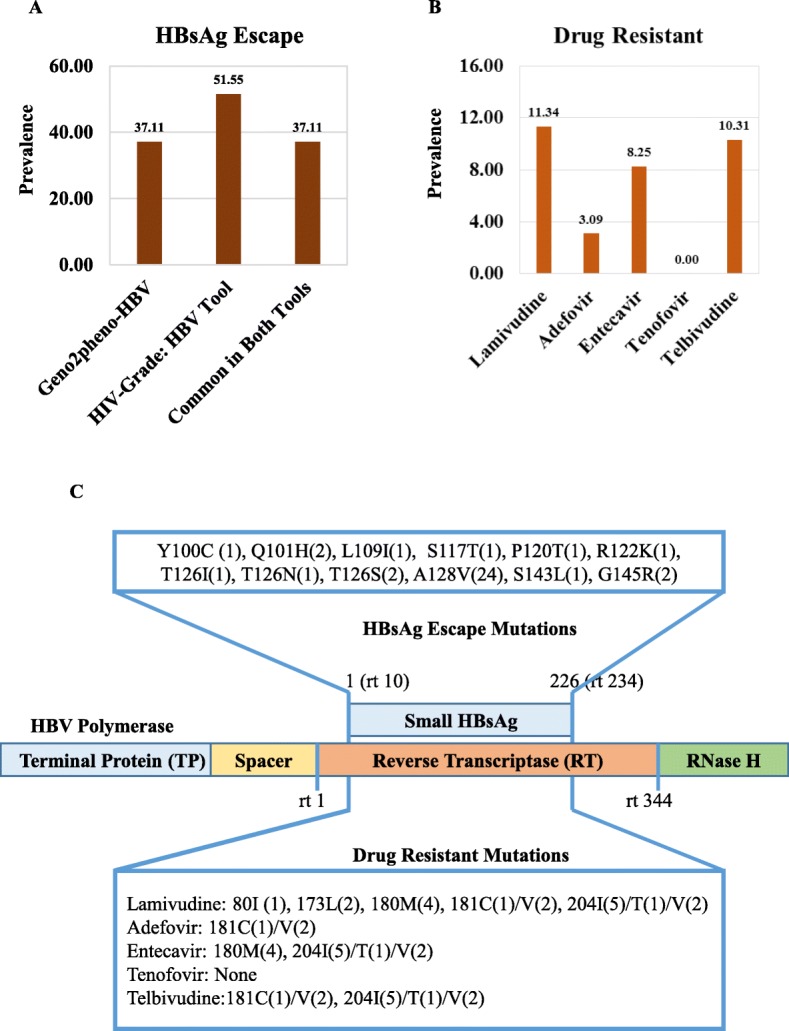
Prevalence of HBsAg escape and drug-resistant mutations in RT/HBsAg overlapping region. Mutations related to HBsAg escape and drug resistant of RT/HBsAg overlapping region were determined by using Geno2pheno-HBV and HIV Grade: HBV-Tool. **a** Overall prevalence of HBsAg escape (**a**) and drug-resistant mutations (**b**) circulating in Bangladesh from 2005 to 2017 determined by two different bioinformatics tools. **c** Specific mutations with their frequency related to HBsAg escape and drug-resistant in the RT/HBsAg overlapping region. The numbers within the bracket showed the frequency of that mutation

The prevalence of HBsAg escape mutation among the 97 sequences with Geno2pheno-HBV and HIV-Grade:HBV-Tool is 37.11 and 51.55%, respectively (Fig. 3a). This variation is because Geno2pheno-HBV could not be able to detect T123A, I126T, and P127T as HBsAg escape. However, the most common vaccine escape HBsAg mutant is 128 V. Mutants 120 T, 126 N, and 145R are responsible for the vaccine, therapy (IG), and detection failure. The mutant 100C and 122 K are responsible for detection failure. The mutant 126I and143L are responsible for vaccine/therapy (IG) and vaccine/detection, respectively (Fig. 3c).

### Analysis of antiviral drug-resistant mutations in RT/ HBsAg overlapping region

Many drug resistant mutations are found within the RT/HBsAg overlapping regions [18]. Therefore, we analyzed the anti-HBV drugs resistance of all 97 RT overlapped HBsAg coding sequence isolated from Bangladesh from 2005 to 2017 based on the Geno2pheno-HBV and HIV-Grade:HBV-Tool. However, results of antiviral drug-resistant mutation in this region from both tools (Geno2pheno-HBV and HIV-Grade:HBV-Tool) are similar with negligible difference, and only the results of Geno2pheno-HBV were described here. The overall prevalence of drug-resistant HBV is 11.34% (11/97) where nine (9) sequences showed resistance to at least three anti-HBV drug among lamivudine, adefovir, entecavir, and telbivudine (Fig. 3b). One sequence showed resistant to four drugs lamivudine, adefovir, entecavir, and telbivudine and one showed resistance against only lamivudine. Tenofovir is found to be susceptible for all 97 sequences. However specific drug resistance prevalence is 11.34% (11/97), 10.30% (10/97), 8.24% (8/97), and 3.09% (3/97) against lamivudine, telbivudine, entecavir, and adefovir, respectively (Fig. 3b).

### Relationship of HBsAg escape and drug-resistant mutations with genotype and serotype

**Fig. 4:**
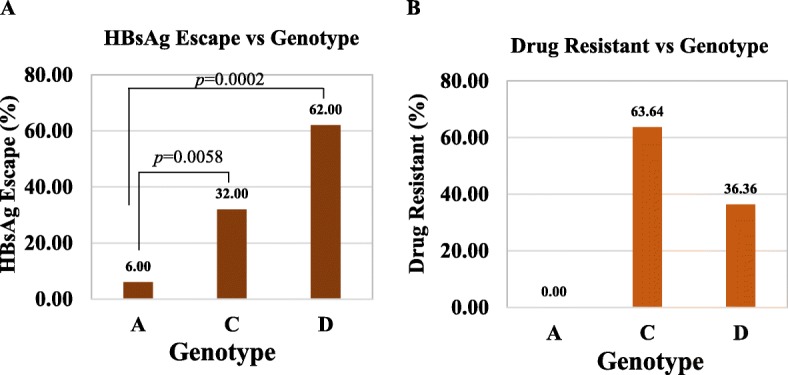
Distribution (Relationship) of HBsAg escape and drug-resistant mutations among the genotypes. Relationship of HBsAg escape and drug-resistant HBV mutations circulating in Bangladesh from 2005 to 2017 was determined (**a** and **b**). Chi-square test was performed to determine the statistical significance level and only the *p-*value greater than 0.05 was mentioned

Among the 36 (37.11%) sequences showing HBsAg escape mutations by Geno2pheno-HBV, the highest prevalence of HBsAg mutations was found in genotype D (69.44%, 25/36) followed by genotype C (22.22%, 8/36) and genotype A (8.33%, 3/36). Interestingly most (24/36) of the sequences contained 128 V mutant which all belongs to only serotype *ayw3* (Genotype D) though HBsAg amino acid position 128 is not considered for serotype determination (Fig. 4a) [25]. Similar results were found in case of HIV-Grade:HBV-Tool analysis, as HBsAg escape mutations in genotype D (62%, 31/50) followed by genotype C (32%, 16/50) and genotype A (6%, 3/50). The statistical analysis showed that drug-resistant mutations are the highest in genotype C (63.64%, 7/11) followed by genotype D (36.36%, 4/11) with absence in genotype A (Fig. 4b). In conclusion, HBsAg escape and drug-resistant mutations are predominant in genotype D (serotype *ayw*) and genotype C (serotype *adr*), respectively, in Bangladesh. These HBsAg escape or drug-resistant mutations relationship with genotype should correspond with HBV subtypes, as there was a strong correlation of genotypes with the serotype (Subtypes) (Fig. 2).

### Relationship between HBsAg escape and drug resistance

**Fig. 5:**
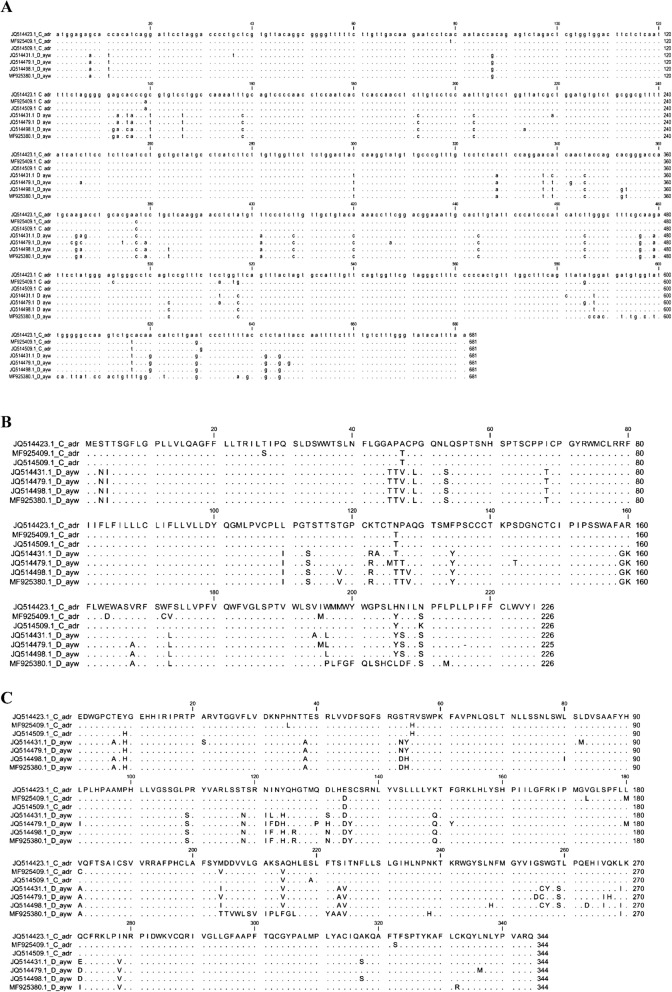
Alignment of the nucleotide sequences (**a**) and amino acid sequences of HBsAg, (**b**) and Pol RT domain (**c**) of the sequences showed both HBsAg escape and drug-resistant mutations

Though 36 or 50 sequences among 97 contained HBsAg escape mutations, we found only 11 or 10 sequences showing drug-resistant mutations by Geno2pheno-HBV and HIV Grade:HBV-Tool, respectively. Only two (2) sequences have HBsAg escape mutations (128 V) among eleven (11) shown to be resistant against anti-HBV drugs (204I/T and 80I/) by Geno2pheno-HBV. On the other hand, six (6) sequences are common having both HBsAg escape and drug-resistant mutations analyzed by HIV Grade:HBV-Tool. One sequence (JQ514498.1) was common in both tools. Therefore, in extend, we analyzed whether the HBsAg escape and drug resistant mutations in these 7 sequences are found from the same nucleotide substitutions. Results demonstrated that none of these mutations are found due to same nucleotide substitutions. However, HBsAg escape and drugs resistant mutations are T123A, I126N/T, P127T, A128V and L80I, L180 M, A181V, M204I/T/V, respectively, found in these common sequences (Fig. 3c and Fig. 5).

## Discussion

Hepatitis B caused by HBV is an important occupational health hazard. According to WHO, 887 thousand people died in 2015 by liver cirrhosis and HCC induced by HBV. HBV associated HCC has been also reported in Bangladesh and patients infected with genotype C hold the greater risk of HCC development [28, 29]. The HBV genotypic prevalence in Bangladesh was performed scatteredly within a limited time slot [22–24]. In this current meta-analytical review analysis of complete S gene sequence coding, RT/HBsAg overlapping region based genotyping from NCBI GenBank from 2005 to 2017 showed that genotype C that is predominant (46.39%) in Bangladesh correlates with previous studies [22, 23]. Genotype-serotype analysis of Bangladeshi HBV sequences showed a strong correlation such as 95.56, 94.44, and 100% of genotypes C, A, and D belong to subtypes *adr*, *adw*, and *ayw*, respectively [30]. The nucleotides diversity determines the HBV genotype and genotype-specific primers designing followed by PCR can differentiate specific genotypes [23, 31, 32]. Whereas the HBV serotype (subtype) is based on the amino acid positions 122 and 160 of HBsAg [33, 34]. Therefore, this strong correlation between HBV genotype-serotype will be very helpful to predict the subtype by performing PCR only as well as redesigning of diagnostic kit and vaccine strain. Regular analysis and detection of currently circulating HBsAg escape mutations is very important for diagnostic failure (diagnostic escape), vaccination escape, and inefficient immunoglobulin (HBIG) therapy [35–38]. Almost all of the diagnostic and/or vaccine escape mutations are found in the MHR because of containing highly conformational B-cells epitope cluster which is the major earmark of neutralizing antibodies to HBsAg [10, 39]. Recently developed tools Geno2pheno-HBV and HIV-Grade:HBV-Tool are very authentic for the analysis of HBsAg escape and drug-resistant mutations of HBV genome sequence [26, 27]. In our analysis, using these two HBV tools, the prevalence of HBsAg escape mutations in Bangladesh among the 97 sequences from 2005 to 2017 is 37.11% or 51.55%. Almost all of the HBsAg escape mutations are secondary mutations associated with vaccine or diagnostic escape. Patients harboring HBV infection showing false-negative diagnosis are more harmful and lead to the development of chronic carrier followed by HCC which demands the establishment of new diagnostic kits [40]. Around 2–5% of the total population in Bangladesh are suffering with chronic HBV infection which require extensive treatment cost [41]. The commonly anti HBV drugs used in Bangladesh are Lamivudine, Adefovir. Entecavir, Tenofovir, and Telbivudine [41]. In our analysis, all of these drugs are resistant at different levels except Tenofovir. Though 8 sequences (8 out of 11 resistant sequences) showed partially resistance against entacavir, only 3 sequences resistant to Adefovir. Lamivudine (11/11) and Telbivudine (10/11) were found to be resistant frequently. As no sequence contained Tenofovir resistant mutations, the treatment strategy needs to replace with Tenofovir instead of others in Bangladesh.

Our prime goal of this review analysis to find out whether there is any statistical relationship between the HBsAg escape and drug-resistant HBV mutations that belong to specific genotype or serotype. Though predominant genotype in Bangladesh is C, higher rate of HBsAg escape mutations were found in genotype D followed by genotype C and very less in genotype A. The predominant mutation is 128 V found only in genotype D (Serotype *ayw3*) though amino acid position 128 is not considered for serotype determinations [25, 42]. HBsAg 128 V mutation is associated with occult HBV infection found in genotype D [43]. These results would be very useful for the analysis of HBV genome sequences in the future including epitope designing for the diagnostic kit and vaccine development. On the other hand, drug-resistant mutations were highest in genotype C followed by genotype D with no report in genotype A in this analysis. However, there were no significant differences or correlation among the HBsAg escape and drug-resistant mutations.

In summary, there is a strong correlation between HBV genotype and serotype with the prevalence of genotypes C, D, and A is 46.39, 35.05, and 18.56%, respectively, in Bangladesh. The genotypes A, C, and D correspond to subtype *adw*, *adr*, and *ayw*, respectively. HBsAg escape mutations were found around 51% in Bangladeshi HBV isolates among which 62% in genotype D followed by 32% in C and only 6% in A whereas drug-resistant mutation is common in genotypes C and D. Sequences showing Lamivudine resistant mutations are higher and Tenofovir is susceptible for all sequences in Bangladesh.

## Conclusion

A strong correlation found in this meta-analysis among the HBV genotype/serotype and HBsAg escape and/or drug-resistant mutations that would be very helpful for genotype-serotype prediction by PCR-based diagnosis and development of vaccine and/or diagnostic kits, and the treatment against HBV infection in future. Drug-resistant mutational analysis supports the use of tenofovir for HBV treatment in Bangladesh.

## Additional file

**Additional file 1.** List of accession numbers of the sequences downloaded from NCBI GenBank and used for analysis in this study. The HBsAg escape and drug-resistant mutational analysis were performed using Geno2pheno-HBV and HIV-Grade:HBV-Tool.

## Data Availability

All the data analyzed in this meta-analysis has been downloaded from the free online database NCBI GenBank which are included in this published article and its supplementary information file.

## Abbreviations

DNA: Deoxyribonucleic acid
ELISA: Enzyme-linked immunosorbent assay
HBIG: Hepatitis B immunoglobulin
HBsAg: Hepatitis B surface protein
HBV: Hepatitis B virus
HCC: Hepatocellular carcinoma
MHR: Major hydrophilic region
NCBI: National Center for Biotechnology Information
PCR: Polymerase chain reaction
